# Case Report: Myelin Oligodendrocyte Glycoprotein Antibody-Associated Relapse With COVID-19

**DOI:** 10.3389/fneur.2020.598531

**Published:** 2020-11-25

**Authors:** Mark Woodhall, James W. Mitchell, Emily Gibbons, Sarah Healy, Patrick Waters, Saif Huda

**Affiliations:** ^1^Nuffield Department of Clinical Neurosciences, University of Oxford, Oxford, United Kingdom; ^2^Department of Neurology, Walton Centre NHS Foundation Trust, Liverpool, United Kingdom; ^3^Institute of Systems, Molecular and Integrative Biology, University of Liverpool, Liverpool, United Kingdom

**Keywords:** myelin-oligodendrocyte glycoprotein (MOG), optic neuritis, Devic's disease, visual loss, post-infectious, autoimmune diseases

## Abstract

A 39-year-old lady with relapsing myelin oligodendrocyte glycoprotein antibody (MOG-IgG) associated disease developed coryzal symptoms, malaise, sweating, and postural dizziness. Six days later she presented with painful progressive right visual loss consistent with optic neuritis. COVID-19 was confirmed by nasopharyngeal swab and MOG-IgG serological reversion was noted. Visual function improved following steroids and plasma exchange. This case highlights a possible causal association between inflammation due to COVID-19 and a relapse of MOG-IgG associated disease. It also highlights the clinical relevance of reporting MOG-IgG titers in MOG-IgG associated disease.

## Introduction

In December 2019, SARS-CoV-2 was identified as the novel human betacoronavirus causing COVID-19, an illness which has since evolved into a pandemic (1). Whilst primarily causing a respiratory illness, reports of neurological manifestations including Guillain-Barré syndrome, encephalitis, and cerebrovascular disease are emerging (2). Herein, we present a relapse of MOG-IgG associated disease (MOGAD) 6 days after presentation with COVID-19 symptoms.

## Case Description

**Figure 1:**
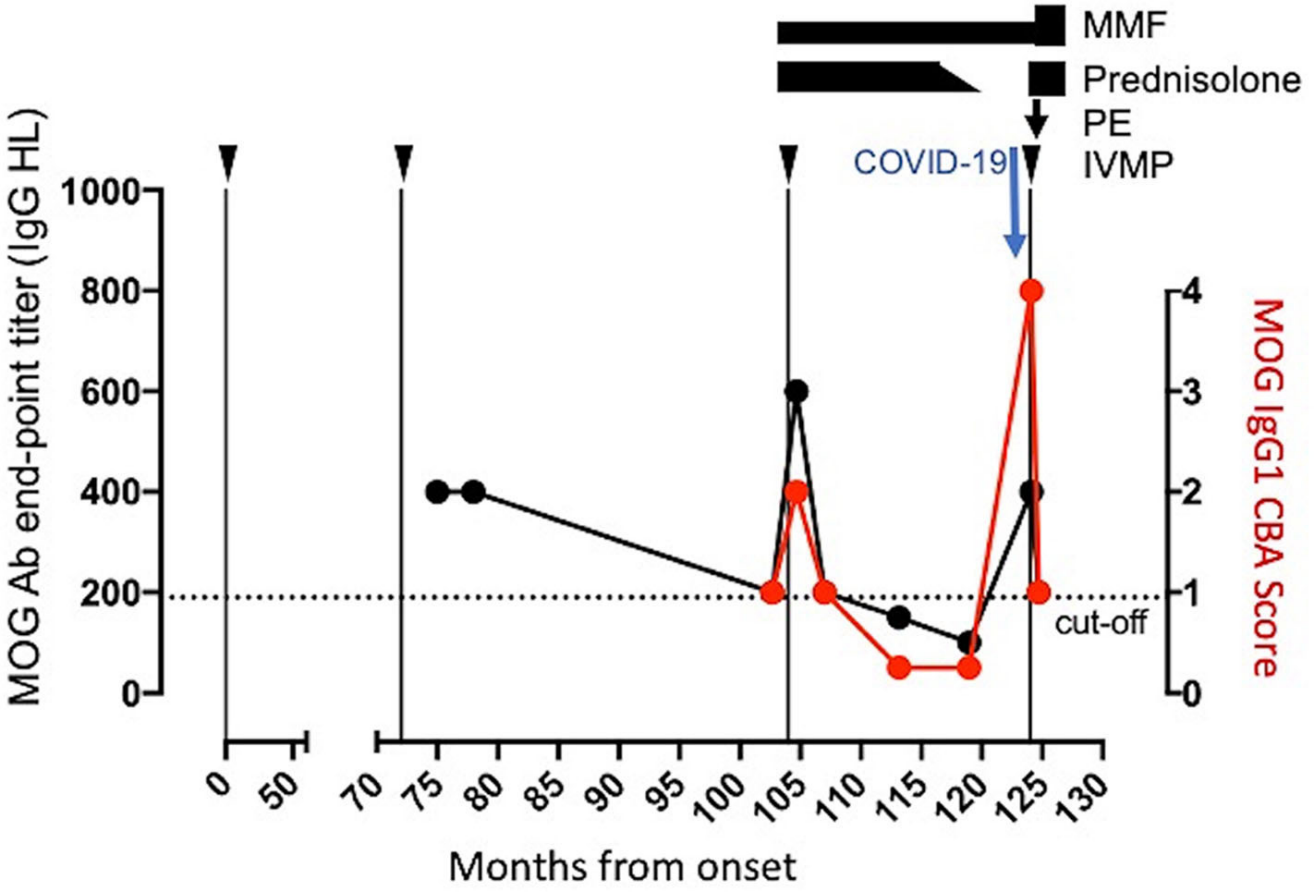
Clinical and serological time course. Arrow heads denote relapses. MMF, mycophenolate mofetil; PE, plasma exchange; IVMP, intravenous methylprednisolone; MOG-Ab, myelin oligodendrocyte glycoprotein antibodies.

A 39-year-old woman developed steroid responsive bilateral optic neuritis in January 2010. Further steroid responsive episodes of unilateral optic neuritis occurred in February 2016 and September 2018. Cerebrospinal fluid (CSF) was acellular with normal protein and absence of unmatched oligoclonal bands and MRI appearances of the brain and cervical cord were normal with no evidence of demyelination. She was treated with mycophenolate mofetil (MMF; 2 g/day) and prednisolone (10 mg/day). She tested positive for serum MOG-IgG1 antibodies by live cell-based assay after her second relapse, confirming a diagnosis of MOGAD and remained persistently positive for more than 3.5 years before MOG IgG1 became negative and MOG-antibody titers fell below the cut-off of 1:200 (Figure 1). At this time, visual acuities were documented as 20/20 (right) and 20/16 (left) with preserved color vision bilaterally. Due to side-effects, corticosteroids were tapered to cessation over 12 weeks, but she remained on MMF (Figure 1). Four months later, she developed malaise, coryzal symptoms, sweating, and postural dizziness. Based on systemic symptoms and a SARS-CoV-2 PCR positive nasopharyngeal swab, COVID-19 was confirmed. Six days later she reported pain on right eye movement with worsening visual acuity. On admission, consistent with optic neuritis she had a right relative afferent pupillary defect with right visual acuity reduced to hand movements. Her left visual acuity remained unchanged at 6/6. The remainder of the cranial nerve examination was unremarkable. There were no pyramidal signs and sensory examination and gait were normal.

Admission work-up including hematological, renal, liver parameters, C-reactive protein, and chest x-ray were normal. However, MOG-IgG1 was strongly positive indicating “seroreversion” with an increase in MOG antibody end-point titer to 1:800 (Figure 1). She received intravenous methylprednisolone 1 g/day for 5 days followed by five cycles of plasma exchange. One week after treatment MOG-antibody titers reduced to 1:200 (Figure 1). MMF was increased to 3 g/day and she was recommenced on prednisolone (40 mg/day) with a plan to slowly taper to a maintenance dose of 10 mg/day. Treatment was well tolerated without adverse effects.

At discharge right visual acuity had improved to 20/125 and through telephone assessment, 3 months later she reported her vision to be “60% back to normal,” with continuing improvement. Unfortunately, during the acute illness, it was not possible to obtain cross-sectional imaging of the brain and orbits due to concerns about SARS-CoV-2 transmission. Brain MRI with dedicated orbital views, 2 months following relapse demonstrated progression of right optic nerve atrophy and subtle T2 signal hyperintensity as compared to the prior study in 2016.

## Discussion

Immunosuppression is being evaluated as a possible treatment option for immunological complications of severe COVID-19 but may increase susceptibility to SARS-CoV-2 infection (3). In this case, immunosuppression did not appear to adversely affect COVID-19 severity which was relatively mild. It is difficult to speculate on susceptibility to SARS-CoV-2 as infection occurred at the height of UK epidemic. Future studies will hopefully shed light on which immunosuppressants are associated with increased susceptibility and better or worse outcomes with COVID-19.

SARS-CoV-2 viral RNA becomes detectable by PCR on a nasopharyngeal swab as early as the 1st day of symptoms and peaks within the 1st week of illness (4). Based on the timing of symptoms and PCR results, in this case, COVID-19 preceded MOGAD relapse and may have been a trigger. SARS-CoV-2 infection results in a dysregulated interferon response but increased expression of several pro-inflammatory cytokines including IL-1B, TNF, and IL-6 (5). Although the time frame between COVID-19 and MOGAD relapse was short (6 days), the increased expression of pro-inflammatory cytokines is seen as early as day 3 in a longitudinal animal model (5). Relapse in this case coincided with MOG-IgG serological “reversion,” i.e. a sub-threshold titer (<1:200) increased to 1:800. It is likely that the SARS-CoV-2 host response reached a critical threshold sufficient to activate MOG-IgG1 specific B-cell subsets, leading to an increase in serum MOG antibody titers and relapse. CD4+ and CD8+ T cell responses associated with the SARS-CoV-2 virus, could have provided the necessary stimulus for bystander activation and co-stimulation of autoreactive T and B-cells, respectively (6). Infection associated relapses in MOGAD are a recognized feature, and in one large series were present in 20% (7). The cessation of steroids 4 months previous is also important to highlight, particularly when considering relapses were seen in 74% of patients treated with mycophenolate mofetil in a recent review of MOGAD treatment (8).

MOG-antibody negative seroconversion in adults is associated with a lower risk of relapse, but this case raises the issue of what constitutes negative seroconversion (9). A serum MOG antibody cut-off of 1:200 maintains disease specificity as a diagnostic test, but in individuals who have reached this threshold, when do we report them as “seronegative” (10)? In the current case MOG antibodies were below the cut-off, but still detectable. This suggests that when following patients with established MOGAD, the continued presence of MOG antibodies below the cut-off may constitute a risk factor for relapse.

This case highlights the clinical relevance longitudinal monitoring of MOG antibody titers in MOGAD. It also highlights a possible link between COVID-19 and MOGAD relapse. This may have implications for disease exacerbation in other relapsing central nervous system inflammatory disorders.

## Data Availability Statement

The original contributions presented in the study are included in the article, further inquiries can be directed to the corresponding author/s.

## Ethics Statement

Written informed consent was obtained from the individual(s) for the publication of any potentially identifiable data included in this article.

## Author Contributions

JM, SHe, EG, and SHu involved in data collection and clinical care of this patient. All authors contributed to the article and approved the submitted version.

## Conflict of Interest

PW is a co-applicant and receives royalties on patent application WO/2010/046716 entitled “Neurological Autoimmune Disorders.” The patent has been licensed to Euroimmun AG for the development of assays for LGI1 and other VGKC-complex antibodies. PW is a co-inventor on “A Diagnostic Strategy to improve specificity of antibody detection.” MOG antibody tests are performed in his laboratory. PW has received research funding from Euroimmun AG. Work in his lab is supported by the NHS highly specialized services for Neuromyelitis optica, UK. SHu has received research support from NMO-UK. The remaining authors declare that the research was conducted in the absence of any commercial or financial relationships that could be construed as a potential conflict of interest.
